# Effects of Artonin E on Cell Growth Inhibition and Apoptosis Induction in Colon Cancer LoVo and HCT116 Cells

**DOI:** 10.3390/molecules27072095

**Published:** 2022-03-24

**Authors:** Kanyaluck Yangnok, Sukanda Innajak, Ratchawin Sawasjirakij, Wilawan Mahabusarakam, Ramida Watanapokasin

**Affiliations:** 1 Department of Biochemistry, Faculty of Medicine, Srinakharinwirot University, Bangkok 10110, Thailand; kanyaluck.yangnok@g.swu.ac.th (K.Y.); suinnajak@gmail.com (S.I.); 2 Faculty of Medicine, Medical University of Lublin, Aleje Racławickie 1, 20-059 Lublin, Poland; tigatrbl@gmail.com; 3 Department of Chemistry, Faculty of Science, Prince of Songkla University, Hat Yai 90112, Thailand; wilawan.m@psu.ac.th

**Keywords:** colon cancer, artonin E, apoptosis, MAPK

## Abstract

Today, colon cancer is the leading cause of cancer death. In Thailand, colon cancer is the third most common cancer in men and the second in women. Currently, the treatments for colon cancer include surgery, chemotherapy, radiation therapy, immunotherapy, hormone therapy, targeted drug therapy, and stem cell therapy. However, some treatments have side effects for cancer patients, causing unwanted symptoms. In addition, targeted therapy comes with a high cost for patients. Therefore, bioactive compounds might be a good choice for colon cancer treatment. In this study, we investigated the effect of artonin E on apoptosis induction in colon cancer LoVo and HCT116 cells. The concentration ranges of artonin E at 3, 5, 10, and 30 µg/mL in LoVo cells and 1, 1.5, 2, and 3 µg/mL in HCT116 cells were examined. The results implied that artonin E decreased cell viability and increased apoptotic cells in a dose-dependent manner. In addition, artonin E stimulated mitochondrial membrane potential (Δ*Ψ*m) changes associated with apoptosis by increasing the sub-G1 population analyzed by flow cytometry. Western blotting showed that artonin E increased the proapoptotic protein, Bax, and decreased anti-apoptotic proteins’ (Bcl-2 and Bcl-x) expression. Moreover, artonin E also increased cleaved caspase-7 and cleaved-PARP expression in both LoVo and HCT116 cells. Interestingly, artonin E induced apoptosis through p-ERK1/2, p-p38/p38, and p-c-Jun expression in both cells. Our results suggested that artonin E induced apoptosis via caspase activation associated with the MAPKs signaling pathway. Therefore, artonin E might be used as a potential anticancer drug for colon cancer in the future.

## 1. Introduction

Cancer is a group of complex genetic diseases in which cells in any part of the body start to grow out of control [1]. These cancer cells can spread into other parts of the body through the bloodstream and lymphatic system [2]. The first place that cancer starts in the body is called the primary site; then, it breaks apart and spreads to a secondary site. These cells begin to grow and form tumors as part of normal tissue [3]. This mechanism is called metastasis, which is considered the main characteristic of cancers [4]. Most cancers are caused by multiple external carcinogenic factors, called environmental factors, combined with internal genetic changes, called genetic factors. All cancers originate from both environment and genetics, due to a combination of multiple external carcinogenic factors combined with internal genetic changes. The carcinogenic agents include UV light, X-rays, viruses, tobacco products, pollutants, chemical reagents, and any sources from outside the body [5]. Furthermore, genetic mutation is a cause of cancer that correlates with long time exposure to the carcinogenic agents, which finally leads to the occurrence of the mutant genes [6]. In fact, most cancers grow slowly, following multiple steps, and take many years to develop. In Thailand, for men, cancer is the third leading cause of death, and it is the second leading cause of death for women [7]. In 2020, the USA reported that the most common type of cancer was breast cancer, while the next most common cancers were lung cancer, prostate cancer, and colon cancer. Colon cancer is caused by an abnormal growth of epithelial cells which form in the lining of the colon or rectum. Treatments for colon cancer include surgery, chemotherapy, radiation therapy, targeted drug therapy, and immunotherapy. The cost of colon cancer treatment is expensive, and it is expected to increase further [8].

Apoptosis, programmed cell death, is a genetically determined process to destroy cells to maintain cellular homeostasis in the tissue. It is considered a vital component of various processes including normal cell turnover, proper development and functioning of the immune system, and embryonic development. In the case of defective apoptotic signals, it may eventually promote carcinogenesis [9].

Artonin E is a prenylated flavonoid that was isolated from the stem bark of the genus Artocarpus. It has been examined in much cancer research [10]. In this study, artonin E was extracted from *Artocarpus elasticus* Reinw. ex Blume found in the Southern part of Thailand, known by the local name, ‘Ka Ok’, ‘Ka O’, or ‘Tue Ka’ [11]. The artonin E was purified by Associate Professor Wilawan Mahabusarakum, Faculty of Science, Prince of Songkla University, Thailand in a previous study. Briefly, *A. elasticus* was immersed in hexane and CH_2_Cl_2_ before being chromatographed on a quick column chromatography and eluted with a solvent of increasing polarity from hexane through acetone, respectively. After that, the extract was purified by Sephadex^TM^ LH-20 column [12]. The artonin E compound is a yellow powder, with a molecular weight of 436.5. Previous studies reported that artonin E has shown efficiency in inhibition of cell growth, proliferation, migration, and invasion and presented a cytotoxic activity leading to apoptosis induction in many cancer cells, such as lung cancer [13], breast cancer [14], ovarian cancer [15], and skin cancer [16]. Our previous study showed that LoVo cells sensitized TRAIL-induced apoptosis in combined treatment with artonin E and TRAIL [17]. However, the effect of artonin E on human colon cancer LoVo and HCT116 cells through the MAPKs signaling pathways has not been reported.

A signaling pathway or cell–cell communication is the basic activity of cells associated with biomolecular interactions. In this study, we investigated the effect of artonin E on mitogen-activated protein kinase (MAPK). MAPKs, serine, and threonine protein kinases can be grouped into three main families of signaling pathways, which modulate physiological and pathophysiological cell responses. Extracellular-signal-regulated kinases (ERKs) are activated by growth factors and survival factors. The ERK pathway is involved in pathogenesis and responds primarily to growth factors and induces cell growth, differentiation, and the oncogenic behavior of human cancers, including lung cancer, breast cancer, and colorectal cancer. When Jun amino terminal kinases (JNKs) and p38, which are activated by environmental stresses and regulate a wide range of cellular processes, are activated, they contribute to cell proliferation, cell differentiation, cell survival, apoptosis, cell inflammation, and cell cycle regulation [18]. In this study, we were interested in investigating the bioactivity of artonin E on apoptosis induction in colorectal cancer cell lines. We aimed to determine the effects of artonin E on the inhibitory effect of cell proliferation, cell growth, and apoptosis induction on the colon cancer LoVo and HCT116 cell lines.

## 2. Results

### 2.1. Artonin E Inhibited Cell Proliferation in LoVo and HCT116 Cells

**Figure 1:**
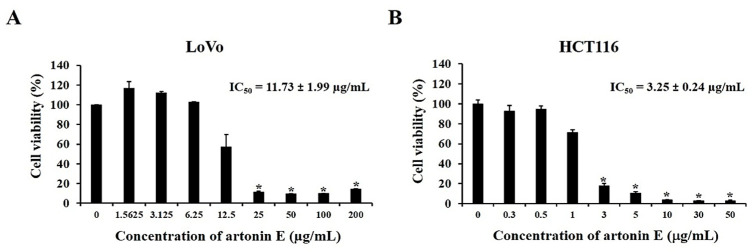
Effect of artonin E on cell viability following treatment with different concentrations of artonin E for 24 h: (**A**) LoVo cells and (**B**) HCT116 cells. The data are presented as mean ± SD (n = 3). * *p* < 0.05 compared with the control group.

An MTT assay was used to determine the antiproliferation activity of artonin E in LoVo and HCT116 cells. The results showed that artonin E inhibited cell proliferation in a dose-dependent manner in both LoVo and HCT116 cells with an IC_50_ value of 11.73 ± 1.99 µg/mL and 3.25 ± 0.24 µg/mL, respectively (Figure 1). Based on the IC_50_ value on each cell line, we chose the concentration of artonin E at 3, 5, 10, and 30 µg/mL for LoVo cells and 1, 1.5, 2, 2.5, and 3 µg/mL for HCT116 cells for the following experiments.

### 2.2. Effect of Artonin E on Apoptosis Induction in LoVo and HCT116 Cells

**Figure 2:**
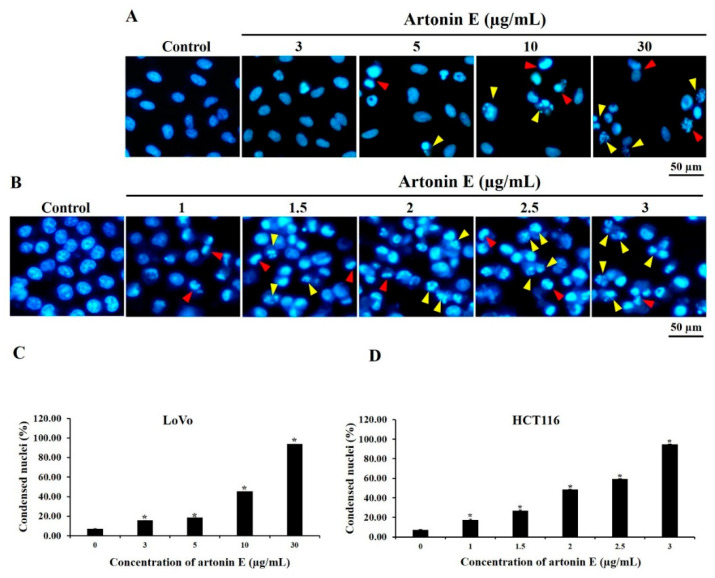
Chromatin condensation and apoptotic bodies in LoVo and HCT116 cells. (**A**) LoVo cells and (**B**) HCT116 cells were treated with various concentrations of artonin E for 24 h; then, chromatin condensation and apoptotic bodies were observed by Hoechst 33342 staining. Red and yellow arrows indicate chromatin condensation and apoptotic bodies, respectively, compared with the control group. Quantification of % nuclear condensation was determined by Hoechst 33342 staining in (**C**) LoVo cells and (**D**) HCT116 cells. * *p* < 0.05 compared with the control group.

Nuclear morphological changes of apoptotic cells were determined by Hoechst 33342 staining, which is a fluorescent dye used for DNA labeling [19]. It preferentially binds to the adenine–thymine (A–T) regions of double-stranded DNA excited by ultraviolet light and emits blue fluorescence at 460 to 490 nm [20]. In this study, the results demonstrated that artonin E induced chromatin condensation and apoptotic bodies in a dose-dependent manner in both LoVo and HCT116 cells (Figure 2).

### 2.3. Mitochondrial Membrane Potential (ΔΨm)

**Figure 3:**
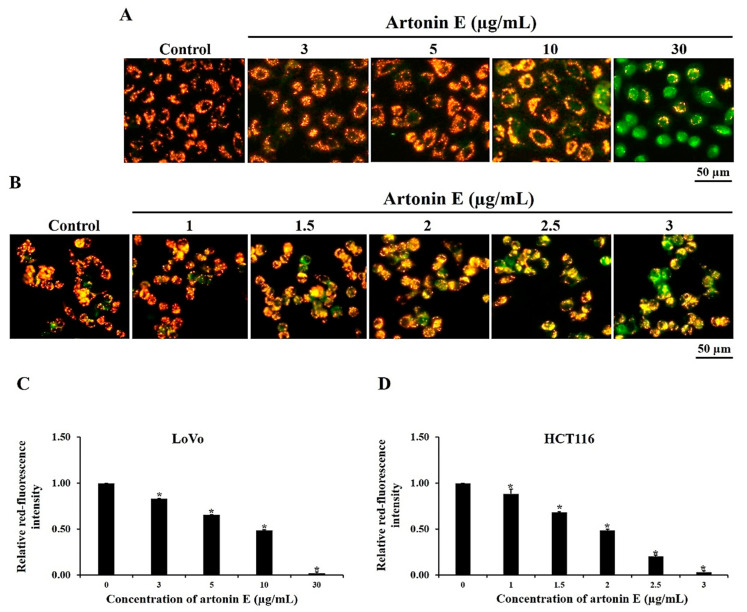
Effect of artonin E on Δ*Ψ*m in LoVo and HCT116 cells. (**A**) LoVo and (**B**) HCT116 cells were treated with different concentrations of artonin E for 6 h. Artonin E decreased red fluorescence and increased green fluorescence in both (**C**) LoVo and (**D**) HCT116 cells, compared with the control cells. * *p* < 0.05 compared with the control group.

During apoptosis, mitochondria were disrupted by proapoptotic protein activation leading to loss of Δ*Ψ*m. For the untreated cells with high Δ*Ψ*m, JC-1 formed J-aggregated complexes as red fluorescence, while apoptotic cells with low Δ*Ψ*m showed green fluorescence. In this study, artonin E induced loss of Δ*Ψ*m in a dose-dependent manner in LoVo and HCT116 cells with decreased red fluorescence and increased green fluorescence compared with the control group (Figure 3). This result implied that artonin E induced apoptosis through mitochondrial damage in both LoVo and HCT116 cells.

### 2.4. Cell Cycle Distribution

**Figure 4:**
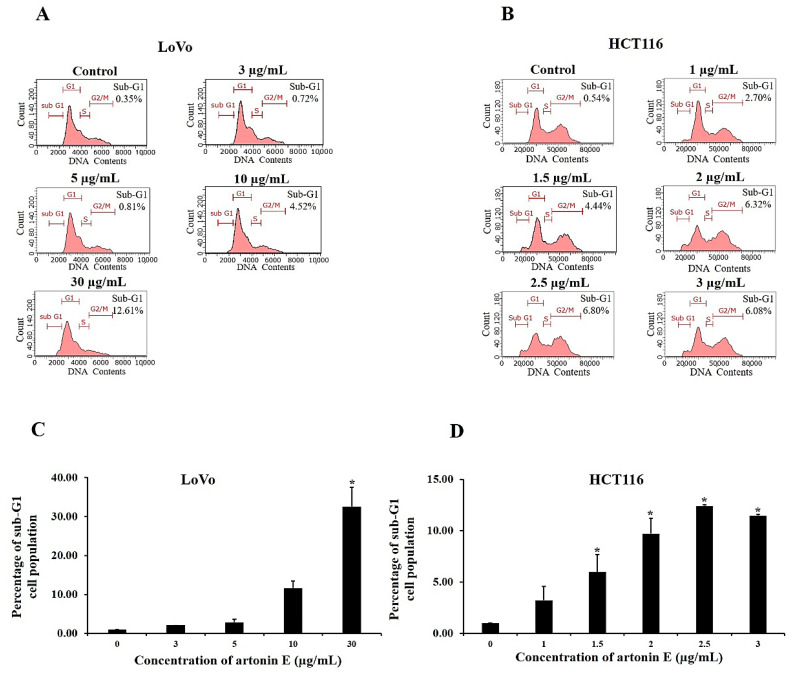
Effect of artonin E on cell cycle distribution. The sub-G1 population was formed with reduced DNA content representing the presence of apoptotic cells. The mean apoptotic population of cells after treatment with artonin E for 24 h for (**A**) LoVo and (**B**) HCT116 cells. Quantification of % cells under the sub-G1 peak of (**C**) LoVo cells and (**D**) HCT116 cells. The data are presented as mean ± SD (n = 3). * *p* < 0.05 compared with the control group.

To confirm the effect of artonin E on apoptosis induction, LoVo and HCT116 cells were stained with PI, and the DNA contents were measured by flow cytometry. LoVo and HCT116 cells treated with 30 µg/mL and 2.5 µg/mL artonin E, respectively, showed a maximum sub-G1 population of 12.61% and 6.80% (Figure 4). In this study, the results revealed that artonin E induced apoptosis in LoVo and HCT116 cells through the accumulation of the sub-G1 population.

### 2.5. Expression of Bcl-2 Family, Caspase-7, and Cleaved-PARP in LoVo and HCT116 Cells

**Figure 5:**
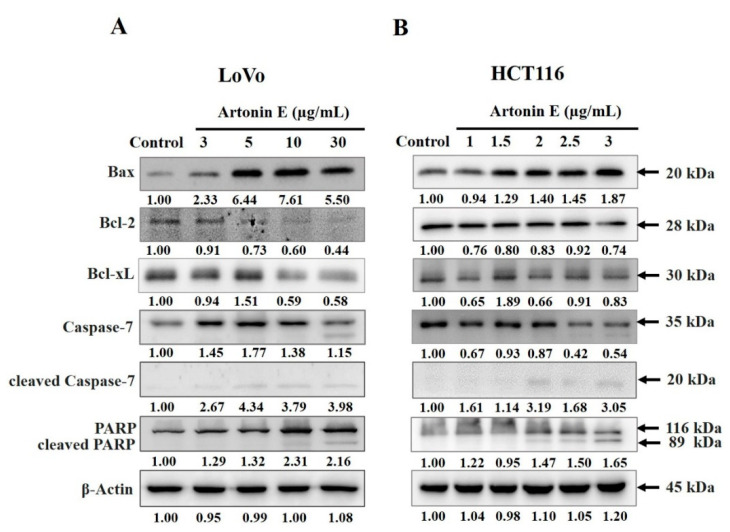
Effect of artonin E on apoptosis protein expression. Cells were treated with different concentrations of artonin E for 24 h. Expression of the Bcl-2 family, caspase-7, and cleaved-PARP in (**A**) LoVo cells and (**B**) HCT116 cells (*p* < 0.05).

Western blotting was performed to detect the activation of apoptosis mediator proteins. The results demonstrated that artonin E increased the level of proapoptotic protein Bax expression and decreased the level of anti-apoptotic proteins Bcl-2 and Bcl-xL in LoVo and HCT116 cells. Moreover, artonin E induced caspase-7 activation and inactivated a DNA repair protein PARP, in both cells (Figure 5). These results indicated that artonin E induced apoptosis associated with caspase-7 activation in both LoVo and HCT116 cells.

### 2.6. Effect of Artonin E on MAPK Protein Expression

**Figure 6:**
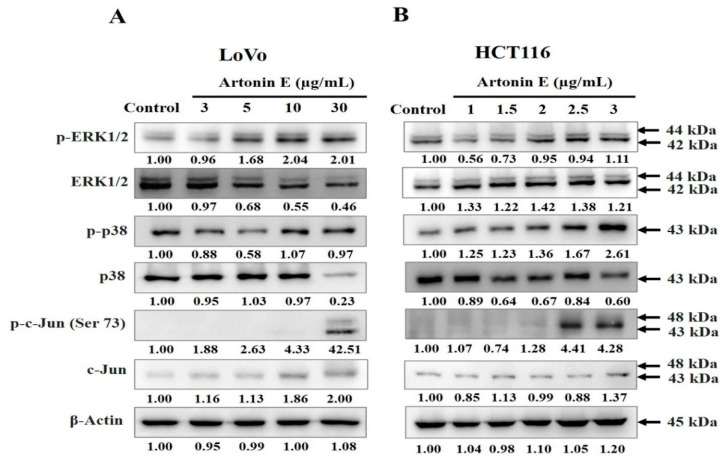
Effect of artonin E on the phosphorylation of MAPK signaling in LoVo and HCT116 cells. Artonin E induced the increased expression of p-ERK1/2, p-p38/p38, and p-c-Jun (Ser73) of (**A**) LoVo cells and (**B**) HCT116 cells, determined by western blot analysis (*p* < 0.05).

MAPK signaling is involved in both apoptosis and cell survival processes. It plays an important role in the transduction of extracellular signals to cellular responses. In this study, we found that artonin E induced increased levels of p-c-Jun, p-ERK1/2, and p-p38/p38 ratios indicating that artonin E induced apoptosis through the MAPK signaling pathway (Figure 6).

## 3. Discussion

Colon cancer is one of the most prevalent and deadly types of cancer in both men and women worldwide. About 90% of patients with metastatic cancer had treatment failure due to acquired resistance to chemotherapy, including resistance to epidermal growth factor receptor (EGFR) antagonists [21]. In contrast, only 10–20% of patients exhibited a primary response to EGFR-targeted therapy, cetuximab, or panitumumab [22,23]. Therefore, more effective treatments with lower side effects for colon cancer need to be discovered. In recent years, the search for novel cancer therapeutics in preclinical trials has turned to natural products with low toxicity and low drug resistance. Previous studies have revealed that artonin E inhibited the growth of SKOV-3 cells and showed less toxicity toward a normal human ovarian cell line T1074 [6]. In addition, artonin E inhibited breast cancer growth and proliferation through the initiation of an intrinsic apoptotic pathway while suppressing an antiapoptotic mechanism, thus circumventing the relative immortality of cancer cells [24]. In this study, the anticancer effect of artonin E was observed in LoVo and HCT116 cells. The results revealed that artonin E could inhibit cell growth in a dose-dependent manner, with an IC_50_ of 11.73 ± 1.99 μg/mL and 3.25 ± 0.24 μg/mL, respectively.

Hoechst 33342 staining was used to confirm apoptosis and nuclear morphological changes of cells through apoptosis induction. These results revealed chromatin condensation and apoptotic bodies after treatment with artonin E at 3, 5, 10, and 30 µg/mL and 1, 1.5, 2, 2.5 m and 3 µg/mL in LoVo and HCT116 cells, respectively. Furthermore, JC-1 staining showed that artonin E stimulated the loss of Δ*Ψ*m, released some proapoptotic proteins from mitochondria to cytosol and induced apoptosome formation, followed by activation of caspase-9 [25]. The active caspase-9 then activated the effector caspase [3,6,7] that cleaves crucial substrates including PARP, resulting in apoptosis induction [26]. These results correlated with a previous study showing condensed chromatin and apoptotic bodies in the epidermoid carcinoma cell line A431 after treatment with artonin E. In addition, the effect of artonin E on the Δ*Ψ*m in A431 cells was observed by increased green fluorescence [27].

The cell cycle is an important mechanism involved with cell growth. During apoptosis, DNA was degraded by cellular endonuclease resulting in less DNA population determined as the sub-G1 peak by flow cytometry analysis compared with healthy cells, [25,28]. Previous studies showed that natural bioactive compounds could inhibit human colon cancer LoVo cells and HCT116 cells by induced sub-G1 population (apoptotic cell population) [29]. In the present results, we revealed that artonin E-treated LoVo and HCT116 cells had an increased sub-G1 population compared with the control cells.

To verify the apoptosis signaling pathway, Bcl-2 family proteins, caspase proteins, and the MAPK signaling pathway were analyzed by Western blotting. There are two main apoptosis signaling pathways, the extrinsic and intrinsic pathways. For artonin E-treated LoVo and HCT116 cells, the results showed increased proapoptotic proteins Bax expression. In contrast, anti-apoptotic proteins Bcl-2 and Bcl-xL were decreased. Moreover, artonin E- increased cleaved caspase-7 and cleaved-PARP expression. These results correlated with the previous study that artonin E showed anti-proliferation and apoptosis induction by induced cleaved caspase-7 and cleaved-PARP activation in A431 skin cancer cells, resulting in apoptosis cell death [26]. In general, there are two types of caspases, initiator and effector caspase; the caspase cascade, caspase-9 (initiator caspase) can activate caspase-7 (effector caspase), which then cleaves vital substrates, such as PARP that plays an important role in DNA repairing [30]. These present results related to previous research demonstrating that artonin E induced apoptosis in several cancer cell types including MDA-MB231, MCF-7, SKOV-3, H460, and A431 [11,15,24,27,31].

Proteins in the MAPK signaling pathway play an important role both in cell survival and cell death [32]. Conventional MAPKs in mammalian cells include ERK1/2, JNK1/2, and p38. ERK1/2 activates Bax and caspase leading to apoptosis. JNK1/2 can activate the transcriptional factors, such as p-c-Jun. p38 is a tumor suppressor, which induces apoptosis and inflammation and inhibits cell proliferation. This result indicated that artonin E induced p-ERK1/2, p-p38/p38 ratio, and p-c-Jun upregulation in LoVo and HCT116 cells leading to apoptosis. In addition, ERK1/2 is important in cell proliferation, cell differentiation, cell growth, and cell survival; however, we found that artonin E upregulated p-ERK1/2 in LoVo and HCT116 cells correlated with the previous report that ERK1/2 could activate caspase and proapoptotic proteins in the Bcl-2 family leading to apoptosis induction [33]. Therefore, our results showed that artonin E induced apoptosis through the MAPK signaling pathway in LoVo and HCT116 cells. From previous studies and our findings, we confirmed that artonin E showed growth inhibition through apoptosis induction in colon cancer cells. Therefore, we hypothesize that the combination of anticancer drugs and artonin E may be more effective for cancer treatment in the future.

## 4. Materials and Methods

### 4.1. Materials

Artonin E [IUPAC name: 5-hydroxy-8,8-dimethyl-3-(3-methylbut-2-enyl)-2-(2,4,5-trihydroxyphenyl) pyrano (2,3-h) chromen-4-one] was obtained from Associate Professor Wilawan Mahabusarakum, Faculty of Science, Prince of Songkla University, Thailand in purified powder form; the extraction and purification were reported in a previous study [12]. Chemicals for cell viability assay including MTT [3-(4,5-Dimethylthiazol-2-yl)-2,5-diphenyltetrazolium bromide] and Dimethylsulfoxide (DMSO) were purchased from Sigma-Aldrich (St. Louis, MO, USA). The fluorescent dye Hoechst 33342 was purchased from Thermo Fisher Scientific, Inc. (InvitrogenTM, Waltham, MA, USA) and the fluorescent dye JC-1 was purchased from Sigma-Aldrich (Merck KGaA, Darmstadt, Germany). Guava Cell Cycle^®^ reagent and antibodies for Western blotting analysis including β-Actin were purchased from Merck Millipore (Merck KGaA, Darmstadt, Germany). Rabbit monoclonal primary antibodies and anti-rabbit immunoglobulin G horseradish peroxidase-conjugated secondary antibodies were obtained from Cell Signaling Technology (Beverly, MA, USA).

### 4.2. Cell Culture

The human colon cancer cell lines, LoVo and HCT116, were obtained from the American Type Culture Collection (ATCC, Manassas, VA, USA). Cells were maintained in RPMI1640 medium in sterile culture flasks. The medium was supplemented by 10% FBS, 100 U/mL penicillin, and 100 µg/mL streptomycin; after that, the cells were incubated in a humidified atmosphere of 5% CO_2_ at 37 °C. The medium was renewed every 2–3 days.

### 4.3. Cell Proliferation and Cell Viability Assays

The MTT assay was used to determine the cytotoxicity of artonin E. Firstly, LoVo and HCT116 cells were seeded at a density of 7 × 10^3^ and 6 × 10^3^ cells/well and treated with artonin E at 3, 5, 10, and 30 µg/mL and 1, 1.5, 2, 2.5, and 3 µg/mL, respectively, while the control group was treated with DMSO. After 24 h, 0.5 mg/mL MTT solution was added to each well and incubated for 2 h at 37 °C. MTT solution was added to each well and incubated at 37 °C for 2 h, then removed; next, 100 uL of DMSO was added to each well to solubilize water insoluble purple formazan crystals. The absorbance was measured at 570 nm by using a microplate reader (Multiskan Sky Microplate Spectrophotometer, Waltham, MA, USA), and the IC_50_ value was calculated by GraphPad Prism 3.03 (GraphPad Software, Inc., San Diego, CA, USA).

### 4.4. Detection of Nuclear Morphological Changes

Nuclear morphological changes of apoptotic cells were stained by Hoechst 33342, which is a fluorescence dye used for DNA labeling [19]. Firstly, LoVo and HCT116 cells were seeded at a density of 2.2 × 10^4^ cells/well and 5 × 10^4^ cells/well and treated with artonin E at 3, 5, 10 and 30 µg/mL and 1, 1.5, 2, 2.5 and 3 µg/mL, respectively, while the control cells were treated with DMSO for 24 h. After incubation, both cells were stained with 5 µg/mL Hoechst 33342 and incubated at 37 °C. The fluorescence signal was measured at 460 to 490 nm [20]. Cells were investigated under a fluorescence microscope (DP73 Olympus, Tokyo, Japan).

### 4.5. Detection of Mitochondrial Membrane Potential (ΔΨm)

Mitochondrial membrane potential (Δ*Ψ*m) was stained by JC-1, which is a lipophilic cationic dye that can selectively enter into mitochondria and reversibly change the color from green to red as the membrane potential increases. Untreated cells with healthy mitochondria showed brighter red fluorescence intensity, while apoptotic cells showed green fluorescence intensity with low Δ*Ψ*m. LoVo and HCT116 cells were seeded at a density of 3.2 × 10^4^ cells/well and 5 × 10^4^ cells/well and treated with artonin E at 3, 5, 10, and 30 µg/mL and 1, 1.5, 2, 2.5, and 3 µg/mL, respectively, while the control cells were treated with DMSO. After incubation, both types of cells were stained with JC-1 and incubated at 37 °C and detected by a fluorescence microscope (DP73 Olympus, Japan).

### 4.6. Cell Cycle Analysis

To study apoptosis induction via accumulation of the sub-G1 population, flow cytometry was carried out. LoVo and HCT116 cells were seeded at 5 × 10^4^ cells/well and allowed to grow for 24 h. Then, LoVo and HCT116 were treated with artonin E at 3, 5, 10, and 30 µg/mL and 1, 1.5, 2, 2.5, and 3 µg/mL, respectively, while the control cells were treated with DMSO. Upon treatment, both cells were harvested, washed with PBS, fixed with ice cold 70% ethanol, and then kept at 4 °C. After fixation, cells were stained according to the manufacturer’s instructions (Guava Cell Cycle^®^ reagent from Merck KGaA, St. Louis, MO, USA). The DNA content was observed using a Guava easyCyte™ flow cytometer and GuavaSoft™ software version 3.3 (Merck KGaA, St. Louis, MO, USA).

### 4.7. Western Blot Analysis

LoVo and HCT116 cells were seeded at a density of 20 × 10^4^ cells/well and 35 × 10^4^ cells/well, respectively, and treated with artonin E at 3, 5, 10, and 30 µg/mL and 1, 1.5, 2, 2.5, and 3 µg/mL, respectively, while the control cells were treated with DMSO. After incubation, cells were harvested, washed with cold PBS, and lysed in RIPA lysis buffer (50 mM Tris-HCL, pH 7.5, 5 mM EDTA, 250 mM NaCI, 0.5% Triton X-100) supplemented with 100 mM PMSF, and a complete mini protease inhibitor cocktail (Roche Diagnostics GmbH, Mannheim, Germany). The cell lysate was centrifuged at 13,000× *g* for 30 min. The supernatant was transferred to a new microcentrifuge tube, and the protein content was determined by Bradford’s method before loading to the gel. The protein samples were separated by sodium dodecyl sulfate-polyacrylamide gel electrophoresis (SDS-PAGE) and transferred onto PVDF membranes by using the Trans-Blot^®^ Turbo™ Transfer System (Bio-Rad Laboratories, Hercules, CA, USA); then, they were blocked with 5% BSA in 1X PBS at room temperature. The primary antibody was a rabbit immunoglobulin G, specific to the mediator proteins including Bax, Bcl-2, Bcl-xL, caspase-7, cleaved caspase-7, ADP-ribose polymerase (PARP), p-ERK1/2, ERK1/2, p-p38, p38, p-c-Jun (Ser73), and β-actin. Then, the anti-rabbit lgG horseradish peroxidase-conjugated secondary antibodies were added for 1 h at room temperature and detected by chemiluminescence using an enhanced chemiluminescence (ECL) reagent. The emitted light was detected by using a chemiluminescence imaging system (Chemiluminescence using Immobilon^TM^ Western chemiluminescent HRP substrate (Merck KGaA, Darmstadt, Germany)).

### 4.8. Statistical Analysis

Statistical significance was assessed by one-way analysis of variance (ANOVA). Statistical analysis was performed using SPSS statistical software package (version 15; SPSS, Inc., Chicago, IL, USA) and using the software GraphPad Prism 3.03 (GraphPad Software, Inc., La Jolla, CA, USA). The Western blotting band intensity was quantified by an Image J densitometer. The differences were considered statistically significant at *p* < 0.05.

## 5. Conclusions

In conclusion, artonin E induced apoptosis associated with the MAPK signaling pathway by promoting apoptosis cell death through p-ERK1/2, p-p38/p38, and p-c-Jun, and it decreased cell survival simultaneously in both types of human colon cancer LoVo and HCT116 cells. Therefore, in the future, artonin E may be further studied in animal models.

## Data Availability

The datasets created and analyzed during the current study are available from the corresponding author on reasonable request.

## Sample Availability

Samples of the compounds are not available from the authors.
